# *Clostridium acetobutylicum atp*G-Knockdown Mutants Increase Extracellular pH in Batch Cultures

**DOI:** 10.3389/fbioe.2021.754250

**Published:** 2021-10-25

**Authors:** Yu-Sin Jang, Hyeon Jeong Seong, Seong Woo Kwon, Yong-Suk Lee, Jung Ae Im, Haeng Lim Lee, Ye Rin Yoon, Sang Yup Lee

**Affiliations:** ^1^ Division of Applied Life Science (BK21), Department of Applied Life Chemistry, Institute of Agriculture and Life Science (IALS), Gyeongsang National University, Jinju, South Korea; ^2^ Department of Chemical and Biomolecular Engineering (BK21 Plus Program), BioProcess Engineering Research Center, Institute for the BioCentury, Korea Advanced Institute of Science and Technology (KAIST), Daejeon, South Korea

**Keywords:** *Clostridium acetobutylicum*, ATPase, *atpG*, knockdown, extracellular pH

## Abstract

ATPase, a key enzyme involved in energy metabolism, has not yet been well studied in *Clostridium acetobutylicum*. Here, we knocked down the *atpG* gene encoding the ATPase gamma subunit in *C. acetobutylicum* ATCC 824 using a mobile group II intron system and analyzed the physiological characteristics of the *atpG* gene knockdown mutant, 824-2866KD. Properties investigated included cell growth, glucose consumption, production of major metabolites, and extracellular pH. Interestingly, in 2-L batch fermentations, 824-2866KD showed no significant difference in metabolite biosynthesis or cell growth compared with the parent ATCC 824. However, the pH value in 824-2866KD cultures at the late stage of the solventogenic phase was abnormally high (pH 6.12), compared with that obtained routinely in the culture of ATCC 824 (pH 5.74). This phenomenon was also observed in batch cultures of another *C. acetobutylicum*, BEKW-2866KD, an *atpG*-knockdown and *pta-buk* double-knockout mutant. The findings reported in this study suggested that ATPase is relatively minor than acid-forming pathway in ATP metabolism in *C. acetobutylicum.*

## Introduction

*Clostridium acetobutylicum* is a strictly anaerobic, gram-positive bacterium that survives in hostile environments by producing endospores (Shao et al., 2007). *C. acetobutylicum* possesses industrially applicable metabolic properties, notably including the production of organic solvents, such as acetone, butanol, and ethanol (Kwon et al., 2020; Shin et al., 2021). *C. acetobutylicum* produces the solvents through biphasic pathway, which is divided into an acidogenic phase and a solventogenic phase (Shao et al., 2007; Im et al., 2021). During the acidogenic phase, which corresponds to the initial growth phase, most carbon sources are used to produce acetate, butyrate, and carbon dioxide (Jang et al., 2012). As cell growth enters the stationary phase, the metabolism of *C. acetobutylicum* shifts to the solventogenic phase (Lütke-Eversloh, 2014), during which organic acids are re-assimilated, and most of the carbon sources are used to produce butanol, acetone, and ethanol as final products (Jang et al., 2012).

**FIGURE 1:**
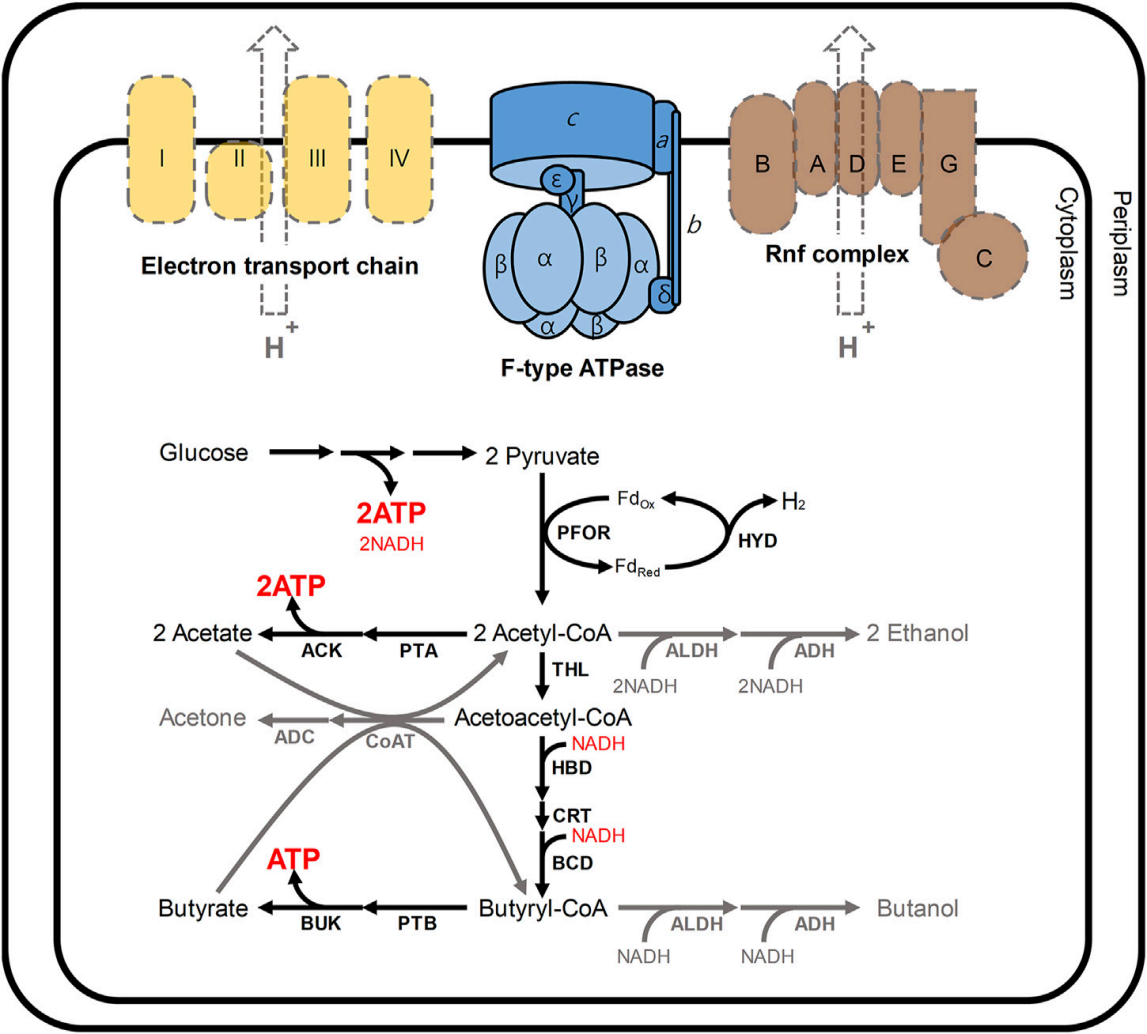
Schematic presentation of the energy and redox metabolism in *C. acetobutylicum.* In *C. acetobutylicum*, F-type ATPase is encoded by the *atp* operon (*atp*IBEFHAGDC). In general, F-type ATPases require the proton motive force to produce ATP; however, neither the electron transport chain (yellow) nor Rnf complex (brown) has been reported in *C. acetobutylicum* (Tremblay et al., 2012). The Rnf complex is commonly reported in other clostridia such as *Clostridium beijerinkii, Clostridium saccharobutylicum* and *Clostridium saccharoperbutylacetonicum*, but it is usual that the Rnf is not found in *C. acetobutylicum* (Poehlein et al., 2017). In this situation, meanwhile, 2 mol of ATP are produced from a glucose through glycolysis. During the initial growth phase (namely, acidogenic phase), additional ATP is produced through the routes for the production of acetate and butyrate. At that time, NADH could be re-oxidized *via* two routes: 1) a cascade reaction for butyrate formation involving 3-hydroxybutyryl-CoA dehydrogenase (HBD) and butyryl-CoA dehydrogenase (BCD); 2) hydrogen production reaction catalyzed by hydrogenase (HYD) coupled with ferredoxin oxidoreductase (PFOR). During solventogenic phase (gray arrows), the function of hydrogenase is turned-off, and NAD^+^ is regenerated by 4 (HBD, BCD, ALDH, and ADH) and 2 (ALDH and ADH) dehydrogenases for butanol and ethanol biosynthesis, respectively. Abbreviations: ACK, acetate kinase; ADC, acetoacetate decarboxylase; ADH, alcohol dehydrogenase; ALDH, aldehyde dehydrogenase; BUK, butyrate kinase, CoAT, CoA transferase; CRT, crotonase; THL, thiolase; PTA, phosphotransacetylase; and PTB, phosphotransbutyrylase.

The reason for this biphasic fermentation is closely related to the energy and redox metabolism in *C. acetobutylicum* (Figure 1) (Jang et al., 2014a; Lütke-Eversloh, 2014). In these bacteria, ATP is primarily produced from glucose through glycolysis (Externbrink et al., 2000). During the initial growth phase in *C. acetobutylicum*, additional ATP is produced through substrate-level phosphorylation, which is coupled to the production of acetate and butyrate (Externbrink et al., 2000). At that time, to regenerate NAD^+^, NADH could be oxidized *via* not only two enzymes 3-hydroxybutyryl-CoA dehydrogenase (HBD) and butyryl-CoA dehydrogenase (BCD) responsible for butyrate formation, but also hydrogenase (HYD) coupled with ferredoxin oxidoreductase (PFOR; Figure 1) (Du et al., 2021; Jiang et al., 2021). As the acidogenic phase progresses, the external pH is continuously lowered to nearby 4.5, and NADH also accumulates, both of which have adverse effects on *C. acetobutylicum* (Jang et al., 2012; Lütke-Eversloh, 2014). At this point, the metabolism of *C. acetobutylicum* shifts from the acidogenic phase to the solventogenic phase (Jang et al., 2012; Li et al., 2020; Thi et al., 2020). After such phase transition, the function of hydrogenase is turned-off, and NAD^+^ is regenerated by 4 and 2 dehydrogenases for butanol and ethanol biosynthesis, respectively (Tremblay et al., 2012; Fast and Papoutsakis, 2018) (see Figure 1 for details). Continuous acid re-assimilation and carbon flux toward solvent production cause the lowered external pH to rise (Kim et al., 2020; Li et al., 2020).

Despite such perfect metabolism for energy and redox regulation through biphasic fermentation, the *atp* operon encoding ATPase was reported in *C. acetobutylicum* genome (Nölling et al., 2001; Cho et al., 2017). The fully sequenced *atp* operon in *C. acetobutylicum* has been shown to include the *atp*IBEFHAGDC (F-type ATPase) (Externbrink et al., 2000). F-type ATPases, which are conjugated to the inner membrane in microbes, generally mediate ATP synthesis through oxidative phosphorylation (Externbrink et al., 2000; Mukherjee and Warshel, 2015; Zharova and Vinogradov, 2017; Kang et al., 2019). F-type ATPases require the proton motive force (PMF) to produce ATP from ADP and inorganic phosphate (Pi); however, neither the electron transport chain nor Rnf complex has been reported in *C. acetobutylicum* (Tremblay et al., 2012; Shin et al., 2021) (Figure 1). Although physiological effects of disrupting ATPase have been analyzed in other organisms, such as *Escherichia coli* (Jensen and Michelsen, 1992; Causey et al., 2003; Shah and Duncan, 2015; Burger et al., 2020), *Lactococcus lactis* (Koebmann et al., 2000; Koebmann et al., 2002a), *Rhodobacter capsulatus* (Borghese et al., 1998), *Saccharomyces cerevisiae* (Weber et al., 1995; Zhang and Zhang, 2019), *Corynebacterium glutamicum* (Sekine et al., 2001; Koch-Koerfges et al., 2012), and *Bacillus subtilis* (Santana et al., 1994), the consequences of ATPase mutation in *C. acetobutylicum* ATCC 824 have not yet been investigated. Here, to reveal the main function of F-type ATPase in *C. acetobutylicum*, we constructed ATPase-knockdown strains and performed a physiological characterization of resulting ATPase-knockdown strains.

## Materials and Methods

### Bacterial Strains, Plasmids, and Culture Conditions

*E. coli* strains and recombinants were grown in Luria-Bertani (LB) broth at 37°C (An et al., 2020; Lone et al., 2020). *C. acetobutylicum* ATCC 824 and the engineered strain BEKW and mutants were grown in clostridial growth medium (CGM) or 2X YTG agar in an anaerobic chamber (Forma Scientific, Marietta, OH, United States) under 4% hydrogen and 96% nitrogen at 37°C (Jang et al., 2012). Ampicillin (50 μg/ml), chloramphenicol (34 μg/ml), or erythromycin (40 μg/ml) was added to the medium, as required.

### Construction of Knockdown Mutants

The mobile group II intron system was used to construct *atpG*-knockdown mutants of *C. acetobutylicum* (Heap et al., 2007; Shao et al., 2007; Jang et al., 2012; Jang et al., 2014b; Kim et al., 2015; Kwon et al., 2020). The *atpG*-targeted intron for knockdown was amplified by overlap extension PCR using the following primers: 2866-IBS, 5′-AAA​AAAG​CTTATA​ATT​ATC​CTT​AAT​AGC​CGA​CCG​TGT​GCG​CCC​AGA​TAG​GGT​G-3′; 2866-EBS1, 5′-CAG​ATTGT​ACAAAT​GTG​GTG​ATA​ACA​GAT​AAG​TCG​ACC​GTG​CTA​ACT​TAC​CTT​TCT​TTG​T-3′; 2866-EBS2, 5′-TGA​ACG​CAA​GTT​TCT​AAT​TTC​GGT​TGC​TAT​CCG​ATA​GAG​GAA​AGT​GTC​T-3′; EBS universal, 5′-CGA​AAT​TAG​AAA​CTT​GCG​TTC​AGT​AAA​C-3′ (Supplementary Table S1). The amplified PCR fragment (∼0.5 kb) was double-digested using restriction enzymes *Bsr*GI and *Hin*dIII, and then ligated into pCACYS3 (Jang et al., 2012) digested using the same enzymes, yielding the recombinant plasmid, pCAC2866KD. Plasmid pCAC2866KD was consecutively transformed into *E. coli* TOP10 (pAN1) containing the plasmid pAN1, which harbors the methyltransferase gene, *φ*3TI (Mermelstein and Papoutsakis, 1993). Thus, the recombinant plasmid, pCAC2866KD, is methylated by the methyltransferase in the resulting *E. coli* strain. *C. acetobutylicum* ATCC 824 and its *pta-buk* double mutant BEKW (Jang et al., 2012) were subsequently transformed with the methylated recombinant plasmid, yielding the *atpG*-knockdown mutant strains, 824-2866KD and BEKW-2866KD, respectively. The resulting *atp*G-knockdown mutants, in which the targeted intron was inserted in the sense strand, were validated by PCR using primers atpG-F and atpG-R (Supplementary Table S1). The intron insertion into the target site on the *atpG* gene was further confirmed by sequencing the DNA fragments obtained from PCR with primers atpG-seq-F and atpG-seq-R using total DNA of the mutant (Supplementary Table S1).

### Batch Fermentation

*C. acetobutylicum* ATCC 824 and its mutants were inoculated into 500-ml Erlenmeyer flasks containing 200 ml CGM and then cultured anaerobically to an optical density at 600 nm (OD_600_) of 1.0 at 37°C (Jang et al., 2012). The resulting seed cultures were transferred into a 5-L Liflus GX bioreactor (Biotron, Gyunggi-do, South Korea) containing 1.8 L CGM for fermentation. The bioreactor was set at an agitation speed of 200 rpm, a nitrogen gas flow rate of 0.25 vvm, and a temperature of 37°C. The pH was automatically maintained above 5.0 with ammonia solution but was not controlled when pH became higher than the set value. Samples were periodically withdrawn from the culture medium for analysis of cell growth and concentrations of glucose, organic acids, and organic solvents.

### Analytical Methods

Samples were collected for monitoring cell growth, glucose consumption, pH, and production of metabolites, including acetate, butyrate, acetone, ethanol, and butanol. Batch fermentations of each strain were independently performed in duplicate. Cell growth was monitored by measuring OD_600_ using an Ultrospec 3000 spectrophotometer (Pharmacia Biotech, Uppsala, Sweden). The concentrations of acetate, butyrate, and glucose were determined using a high-performance liquid chromatography (HPLC) system (Prostar; Varian, Palo Alto, CA, United States) equipped with a packed column (Metacarb 87H; MetaChem Technologies, Torrance, CA, United States) and refractive index detector (RI-27; Shodex, Japan). The mobile phase consisted of 0.01 N H_2_SO_4_ (Im et al., 2019; Chun and Sang, 2020; Lee et al., 2020). The concentrations of acetone, butanol, and ethanol were determined using a gas chromatography system (Agilent 7890; Agilent Technologies, California, United States) equipped with a packed column (80/120 Carbopack BAW glass column; Supelco, Bellefonte, PA, United States) and flame ionization detector (Jang et al., 2012; Baek et al., 2019). Helium gas was used for the mobile phase.

## Results and Discussion

### Construction of the *atpG*-Knockdown *C. acetobutylicum* Mutants

**FIGURE 2:**
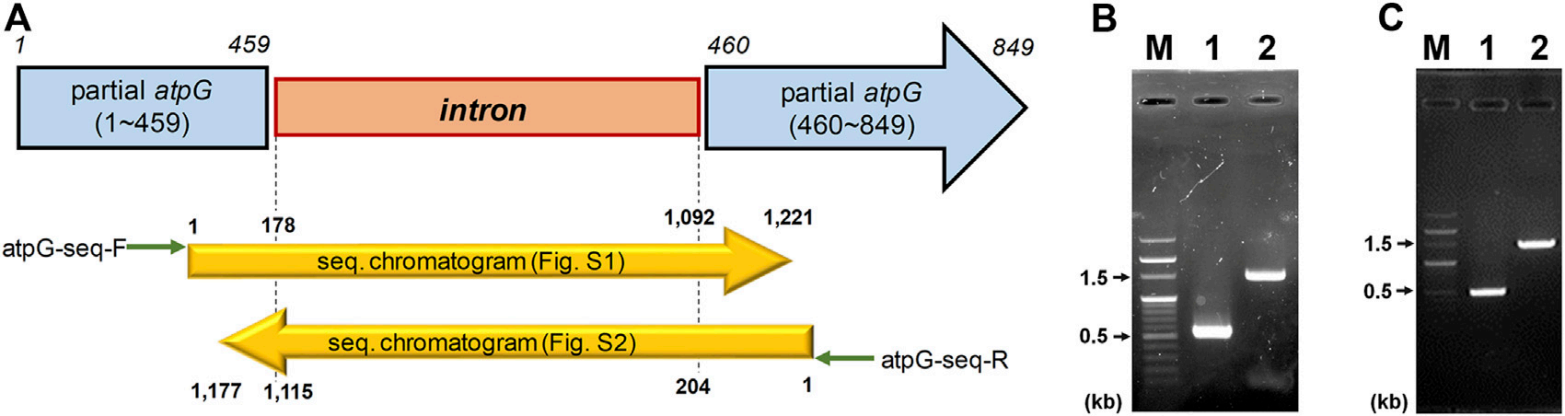
Inactivation of *C. acetobutylicum atpG* gene by the intron insertion using mobile group II intron system. **(A)** Schematic diagram of the mutated *atpG* gene (blue) constructed by intron (orange) insertion. The intron was inserted between 459th and 460th nucleotides in the wild-type *atpG* gene, which was confirmed by sequencing using primers atpG-seq-F (green arrow) and atpG-seq-R (reverse green arrow; Supplementary Table S1). The mutated *atpG* gene was schematically aligned with DNA sequencing chromatograms (yellow arrows; see Supplementary Figures S1, S2 for detailed chromatogram). **(B,C)** Validation of the *atpG* gene mutation in strains 824-2866KD **(B)** and BEKW-2866KD **(C)**. The *atp*G-knockdown mutants, 824-2866KD and BEKW-2866KD were validated by PCR using primers atpG-F and atpG-R (Supplementary Table S1). **(B)** M, 100-bp marker; lane #1, ATCC 824; lane #2, 824-2866KD. **(C)** M, 100-bp marker; lane #1, BEKW; lane #2, BEKW-2866KD.

CAC2866 (*atp*G encoding ATPase gamma subunit), one of nine ATPase-coding genes found in *C. acetobutylicum*, is an important part of the ATPase enzyme (Externbrink et al., 2000). The ATPase gamma subunit forms the central shaft, which forms the connection between the F_0_ rotary motor and the F_1_ catalytic complex (Figure 1; Mukherjee and Warshel, 2015). Disruption of the gamma subunit of F-ATPase in other organisms decreases ATPase activity and ATP levels, resulting in cell growth inhibition and a shift in metabolism (Iwamoto et al., 1990; Shin et al., 1992; Lai-Zhang et al., 1999). Accordingly, to investigate the effects of ATPase knockdown on physiological characteristics of *C. acetobutylicum*, we constructed *atpG*-knockdown mutant strains, 824-2866KD and BEKW-2866KD from wild-type ATCC 824 and the *pta-buk* double mutant BEKW strains, respectively, by using mobile group II intron system (Figure 2; Supplementary Figures S1, S2).

### Effects of *atpG* Knockdown on Cell Growth, Glucose Consumption, and Metabolite Production

**FIGURE 3:**
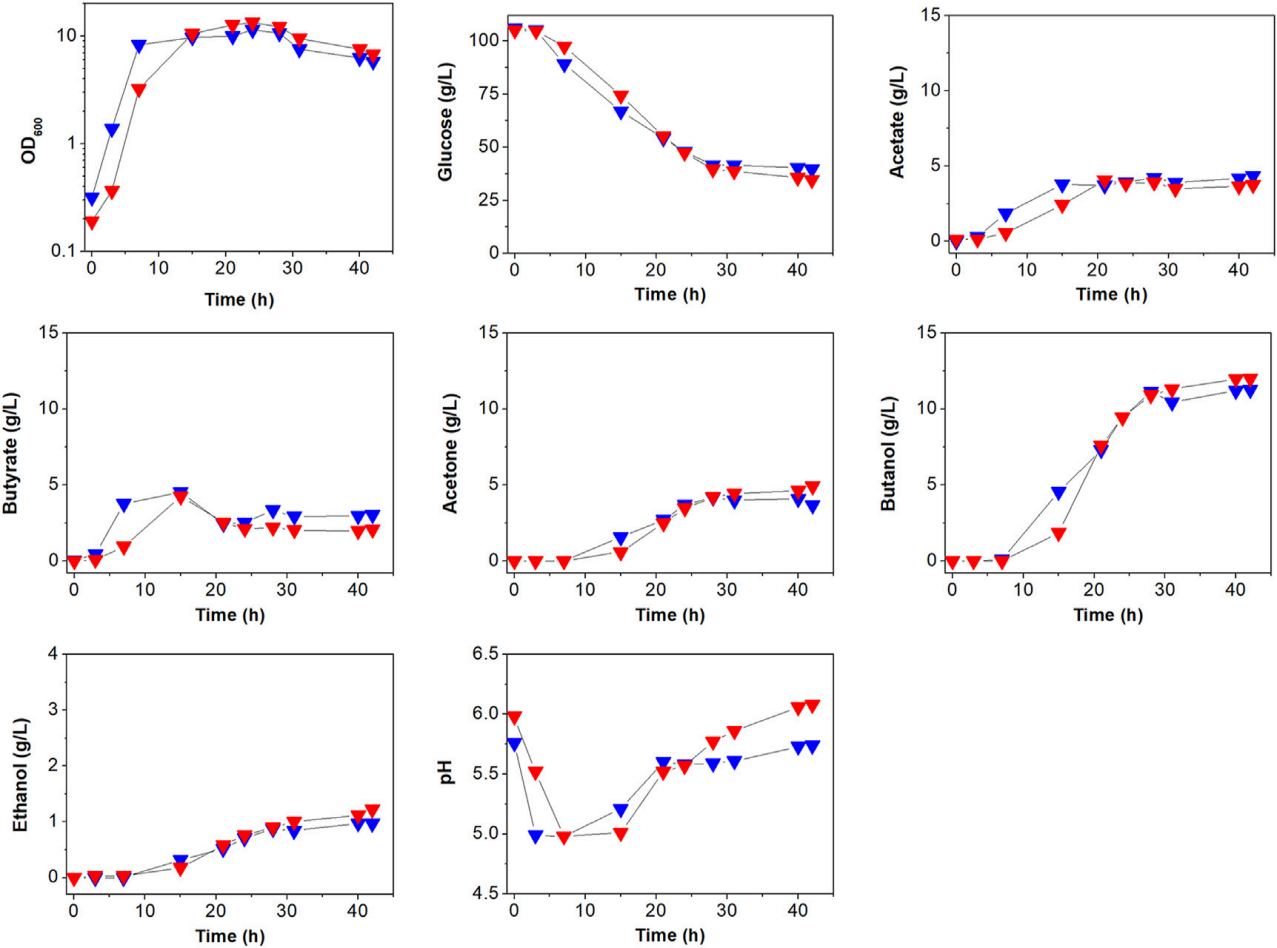
Comparison of batch fermentation profiles between *C. acetobutylicum* ATCC 824 (blue) and its mutant 824-2866KD (red). Fermentation parameters are cell growth (OD_600_) glucose consumption, acids (acetate and butyrate) production, and solvents (acetone, butanol, and ethanol) production. Merged version of batch fermentation profiles of *C. acetobutylicum* 824-2866KD are shown in Supplementary Figure S3A. Other reproduced bioreactor cultivation profiles are shown in Supplementary Figure S3B.

To see the effects of *atpG* knockdown on physiological characteristics, we first analyzed and compared cell growth between ATCC 824 and 824-2866KD (Figure 3; Supplementary Figure S3). There was no apparent difference in growth between ATCC 824 and 824-2866KD (Figure 3), even though disruption of ATPase is known to reduce ATPase activity and ATP level, which in turn inhibits cell growth in non-clostridia strains (Iwamoto et al., 1990; Ferrandiz and De La Campa, 2002; Causey et al., 2003; Cipriano et al., 2006; Kim et al., 2020).

The effects of *atpG* knockdown in *C. acetobutylicum* were also assessed by examining glucose consumption, which is known to be affected by ATP levels (Koebmann et al., 2002b; Dai et al., 2020). Glucose concentration decreased steadily during exponential and stationary phases in both ATCC 824 and 824-2866KD (Figure 3; Supplementary Figure S3). After 28 h, glucose consumption in ATCC 824 was 66.5 g/L and was maintained at 39.5 g/L (Figure 3). 824-2866KD showed a similar decrease in glucose consumption rate, which reached 70.5 g/L at 28 h and was maintained at 34.5 g/L (Figure 3). Thus, these results show no significant changes in glucose consumption in *atpG*-knockdown *C. acetobutylicum* mutant comparing with the parent ATCC 824 strain.

The effects of *atpG* knockdown were further investigated by analyzing the production of metabolites (Figure 3; Supplementary Figure S3). The highest concentrations of acetate and butyrate in 824-2866KD culture were 3.9 g/L and 4.0 g/L, respectively, representing 91.6 and 88.9% of concentrations in ATCC 824 fermentation (Figure 3). During the solventogenic phase, the final concentrations of acetate and acetone in 824-2866KD were also similar to those in ATCC 824 (Figure 3). However, residual butyrate in the fermentation using 824-2866KD was slightly lower than that of the ATCC 824, with a difference of exactly 0.98 g/L at the endpoint (Figure 3). The lack of change (or minor change) in acid and acetone concentrations indicates that acid re-assimilation is also not majorly affected by ATPase knockdown. The production of ethanol and butanol in 824-2866KD culture were 1.4 g/L and 12.4 g/L, respectively, which were also similar to the corresponding concentrations of 1.0 g/L and 11.3 g/L in ATCC 824 fermentation (Figure 3). Previous studies have reported that disruption of ATPase shifts metabolic flux toward byproducts because ATPase-disrupted mutants produce ATP through substrate-level phosphorylation, not by oxidative phosphorylation (Koebmann et al., 2002a; Koebmann et al., 2002b). It seems that as most ATP in *C. acetobutylicum* is produced through substrate-level phosphorylation, the ATPase-knockdown mutant showed no significant changes in acidogenic or and solventogenic phases.

### Effect of *atpG* Knockdown on Extracellular pH

The effect of *atpG* knockdown was also analyzed by comparing extracellular pH between ATCC 824 and 824-2866KD (Figure 3; Supplementary Figure S3). Throughout the entire fermentation period, the bioreactor controller adjusted the external pH to maintain it above 5.0. During the acidogenic phase, ATCC 824 and 824-2866KD reached pH 5.0 and maintained it by adding ammonia solution to avoid decreasing pH values by the production of organic acids. During the subsequent solventogenic phase, pH rose as a result of acid re-assimilation in both ATCC 824 and 824-2866KD cultures (Figure 3). The pH rose steadily after 20 h, reaching pH 5.74 in ATCC 824 culture (Figure 3). On the other hand, pH rose steadily for more than 40 h in 824-2866KD culture, reaching a value of 6.12 at the late stage of the solventogenic phase, a value significantly higher than that in ATCC 824 fermentation (Figure 3). These results show that ATPase activity is affected to the extracellular pH in *C. acetobutylicum* fermentation.

### Effect of *atpG* Knockdown on Physiological Characteristics of *C. acetobutylicum* BEKW

**FIGURE 4:**
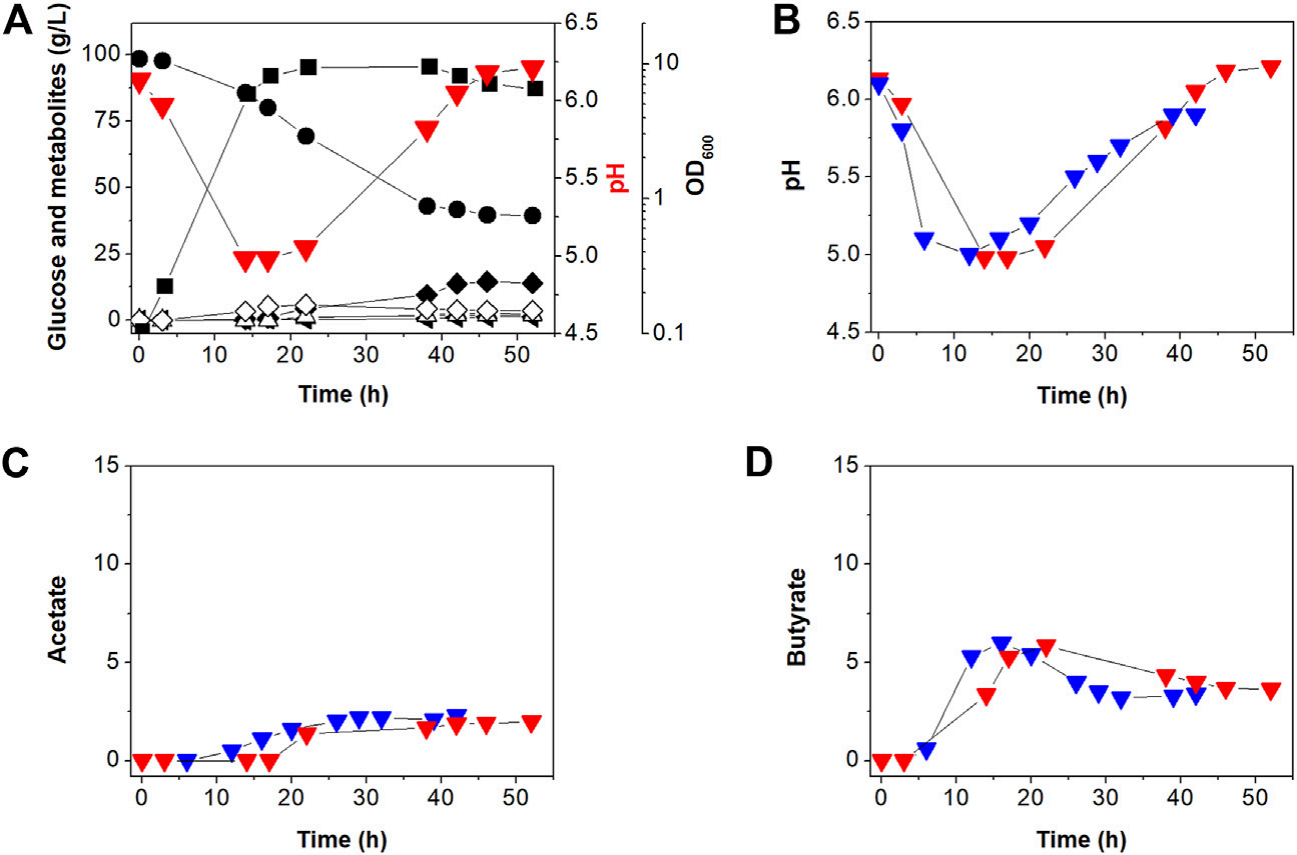
Batch fermentation profiles of *C. acetobutylicum* BEKW-2866KD in bioreactor containing 2-L CGM (A). **(A)** Symbols are: •, glucose; ■, cell density (OD_600_); 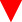, extracellular pH; △, acetate; ◇, butyrate; ▲, acetone; ◂, ethanol; and ◆, butanol. Other reproduced bioreactor cultivation profiles are shown in Supplementary Figure S4. **(B–D)** Comparison of the external pH change **(B)**, acetate **(C)**, and butyrate **(D)** in batch fermentations between *C. acetobutylicum* BEKW (blue) and its mutant BEKW-2866KD (red). For this comparison, pH, acetate, and butyrate values in the parent BEKW fermentation were obtained from our previous work (Jang et al., 2012).

Our previous work (Jang et al., 2012) showed that *C. acetobutylicum* BEKW exhibited higher butanol production (16.0 g/L) than *C. acetobutylicum* ATCC 824 (11.8 g/L). Two enzymes including phosphotransacetylase and butyrate kinase encoded by the *pta* and *buk*, respectively, operate primarily in the acidogenic phase to synthesize the organic acids, acetate and butyrate, respectively, in addition to producing ATP through substrate-level phosphorylation (Lütke-Eversloh, 2014). To determine the effects of *atpG* knockdown in BEKW, we cultured the mutant, BEKW-2866KD in 2-L bioreactor (Figure 4A; Supplementary Figure S4).

First, we compared cell growth and glucose consumption of BEKW-2866KD with that in BEKW. Glucose concentration decreased steadily during exponential and stationary phases in BEKW-2866KD (Figure 4A). Glucose consumption ceased by 38 h and was maintained at 39.91 g/L (Figure 4A). Ultimately, total glucose consumption was 56.60 g, which was not significantly different from that in BEKW (Jang et al., 2012). Consistent with the similar glucose consumption in BEKW and mutant strains, cell growth was also unaffected by *atpG* knockdown (Figure 4A; Jang et al., 2012). Production of the metabolites, acetate, butyrate, acetone, ethanol, and butanol, by BEKW-2866KD, was also analyzed and compared with that of BEKW (Jang et al., 2012). This analysis could be not confirmed significant changes in physiological characteristics (Figure 4). Furthermore, we found that the identified difference in residual butyrate between ATCC 824 and 824-2866KD was not repeated in the comparison between BEKW and BEKW-2866KD (Figure 4D).

The extracellular pH of BEKW and BEKW-2866KD, cultured while maintaining the pH above 5.0, was comparatively analyzed. During the solventogenic phase, pH steadily rose because of acid re-assimilation in both BEKW and BEKW-2866KD fermentations. The extracellular pH in BEKW cultures reached 5.9, a value that was maintained after 39 h (Jang et al., 2012). BEKW-2866KD reach a higher pH value of pH 6.54, which was maintained after 46 h (Figure 4B). In other reproduced bioreactor cultivation, pH 6.89 was observed at 46 h (Supplementary Figure S4). These results are similar to those obtained in comparisons between ATCC 824 and 824-2866KD, described above. The finding that *atpG* knockdown caused no significant differences in cell growth, glucose consumption, or metabolites production indicates that ATPase is relatively minor than acid-forming pathway in ATP metabolism in *C. acetobutylicum*. However, the fact that ATPase knockdown similarly affected extracellular pH in *atpG* knockdown strains indicates that ATPase is affected to extracellular pH at the late stationary phase in *C. acetobutylicum* fermentation. Taken together, it seems that the external pH was affected by not only the residual acids but also other effectors, such as inhibition of proton pumping by ATPase. Depending on the situation, F-ATPase can reversibly synthesize or degrade ATP (Löbau et al., 1998; Bowler et al., 2006; Hayashi et al., 2012). ATP is hydrolyzed to create a proton gradient through the plasma membrane, while PMF is used for ATP synthesis (Costa et al., 2021). The increase in extracellular pH shown in this study is presumed to be due to inhibition of proton pumping across the membrane by knockdown of the *atpG* gene. This seems to be closely related to the recent report that ATPase is inhibited by butanol, which resulted in a low intracellular pH and reduction of PMF (Costa et al., 2021).

In this study, we first constructed the *atpG* knockdown strains using the mobile group II intron system to investigate the role of the ATPase in *C. acetobutylicum*. Although other ATPase-disrupted non-clostridia organisms show prominent differences in ATP synthesis and cell growth, the *atpG* knockdown mutants of *C. acetobutylicum* ATCC 824 and BEKW, 824-2866KD and BEKW-2866KD, respectively, showed no significant changes in physiological characteristics except extracellular pH. The inference is that most ATP is produced through substrate-level phosphorylation in glycolysis and the acid-forming pathways in *C. acetobutylicum*. Detection of the ATP level may help to explain the phenomenon found in this work. As ATP and redox metabolism is complexly combined to biphasic fermentation in *C. acetobutylicum*, however, it is needed to approach it with a more elaborate strategy.

## Data Availability

The original contributions presented in the study are included in the article/Supplementary Material, further inquiries can be directed to the corresponding authors.
