# Supplementary material for: Clostridium acetobutylicum atpG-Knockdown Mutants Increase Extracellular pH in Batch Cultures
Source: Front Bioeng Biotechnol. 2021 Oct 25;9:754250. doi: 10.3389/fbioe.2021.754250 (PMC8573202; doi:10.3389/fbioe.2021.754250)
Supplement: Supplementary file 1 [file DataSheet1.PDF]

Supplementary Material

*Clostridium acetobutylicum atp*G-knockdown mutants increase extracellular pH in batch cultures

Yu-Sin Jang[1,*], Hyeon Jeong Seong[1], Seong Woo Kwon[1], Yong-Suk Lee[1], Jung Ae Im[2], Haeng Lim Lee[1], Ye Rin Yoon[1], Sang Yup Lee[2,*]

[1] Division of Applied Life Science (BK21), Department of Applied Life Chemistry, Institute of Agriculture & Life Science (IALS), Gyeongsang National University, Jinju 52828, Republic of Korea

[2] Department of Chemical and Biomolecular Engineering (BK21 Plus Program), BioProcess Engineering Research Center, Institute for the BioCentury, Korea Advanced Institute of Science and Technology (KAIST), Daejeon, Republic of Korea

*Corresponding authors.

Yu-Sin Jang (jangys@gnu.ac.kr)

Sang Yup Lee (leesy@kaist.ac.kr)

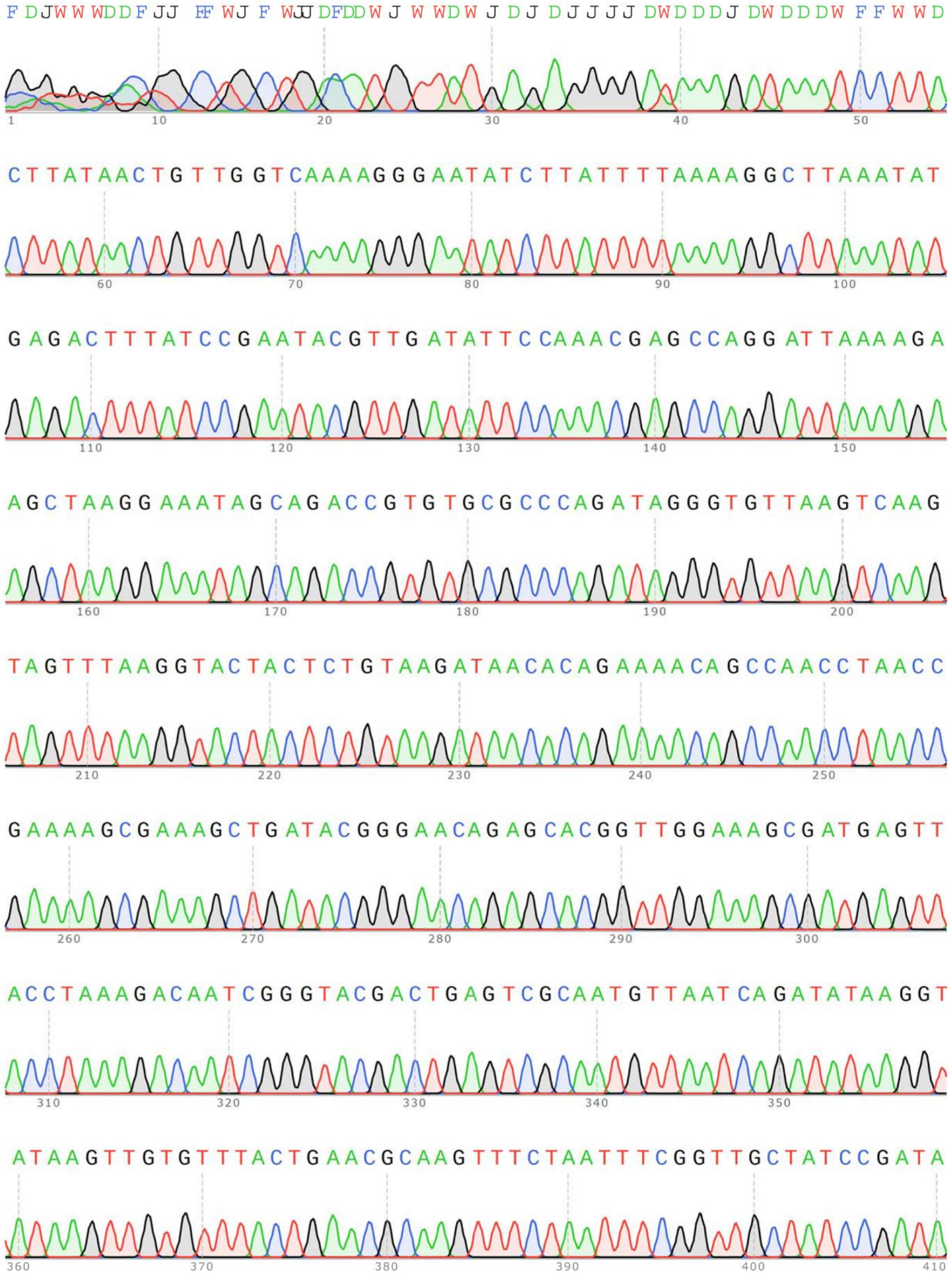

Supplementary Figure S1 DNA sequencing chromatogram for the mutated *atpG* gene in 824-2866KD using atpG-seq-F primer.

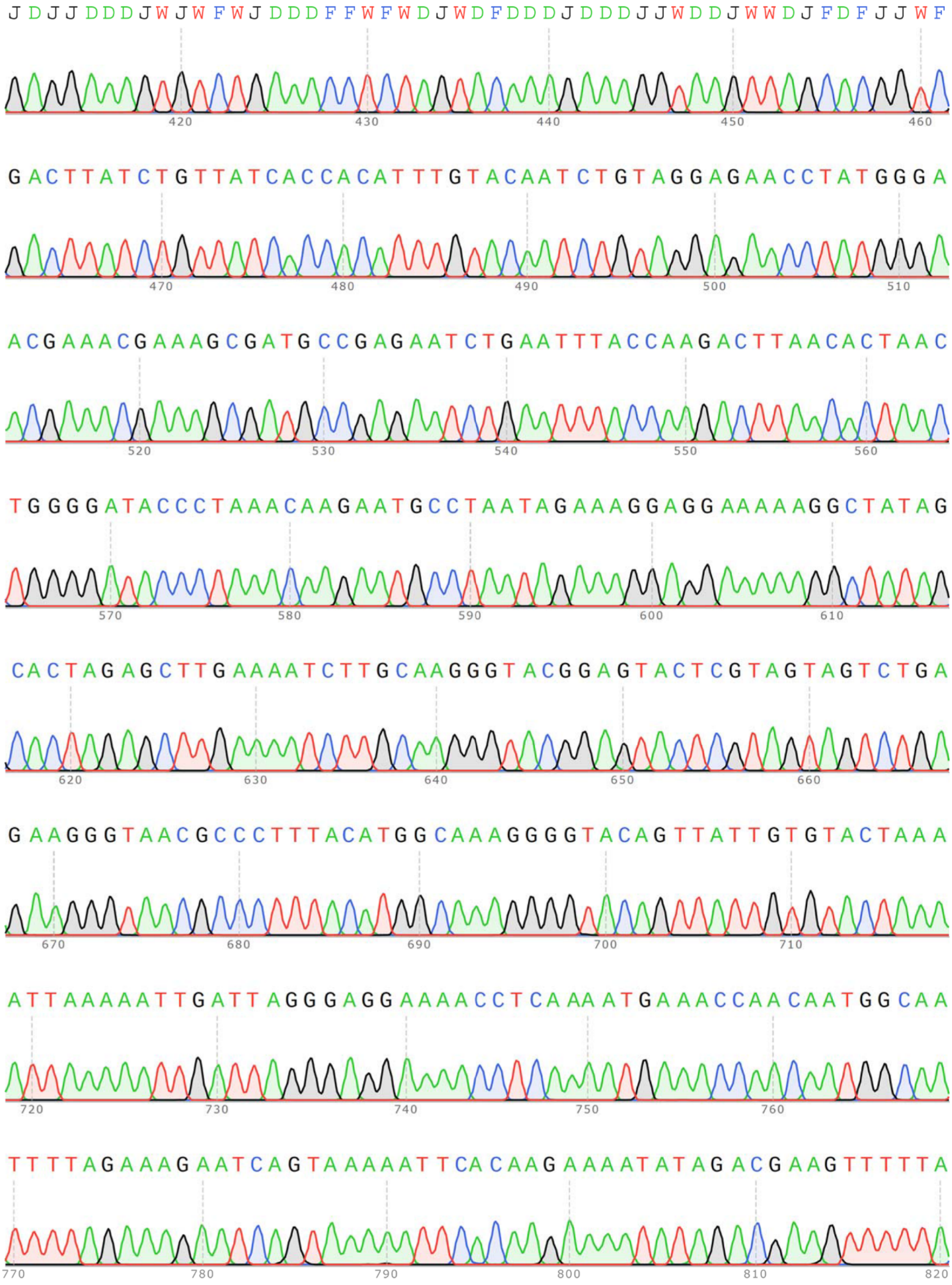

Supplementary Figure S1 (Continued).

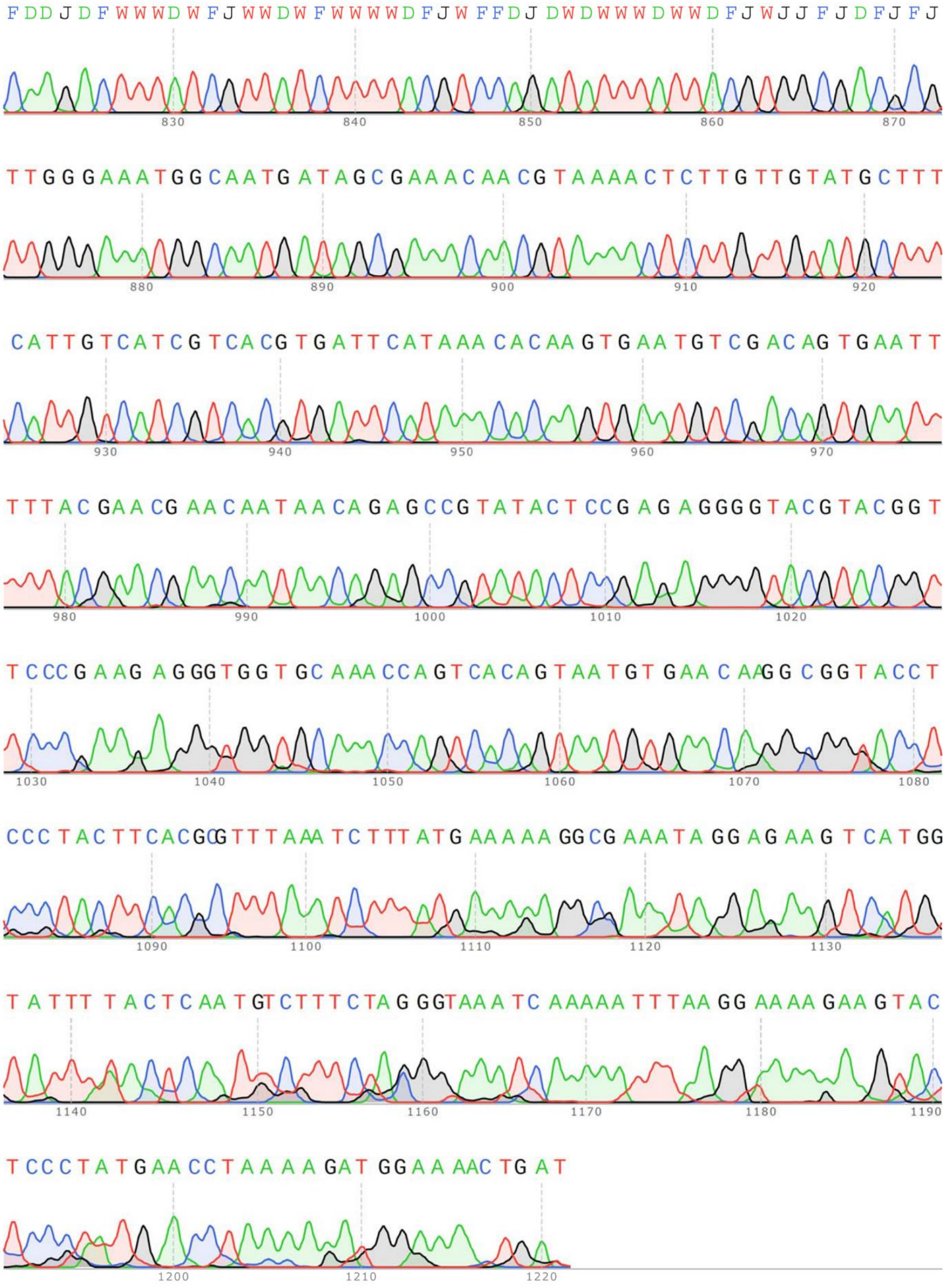

Supplementary Figure S1 (Continued).

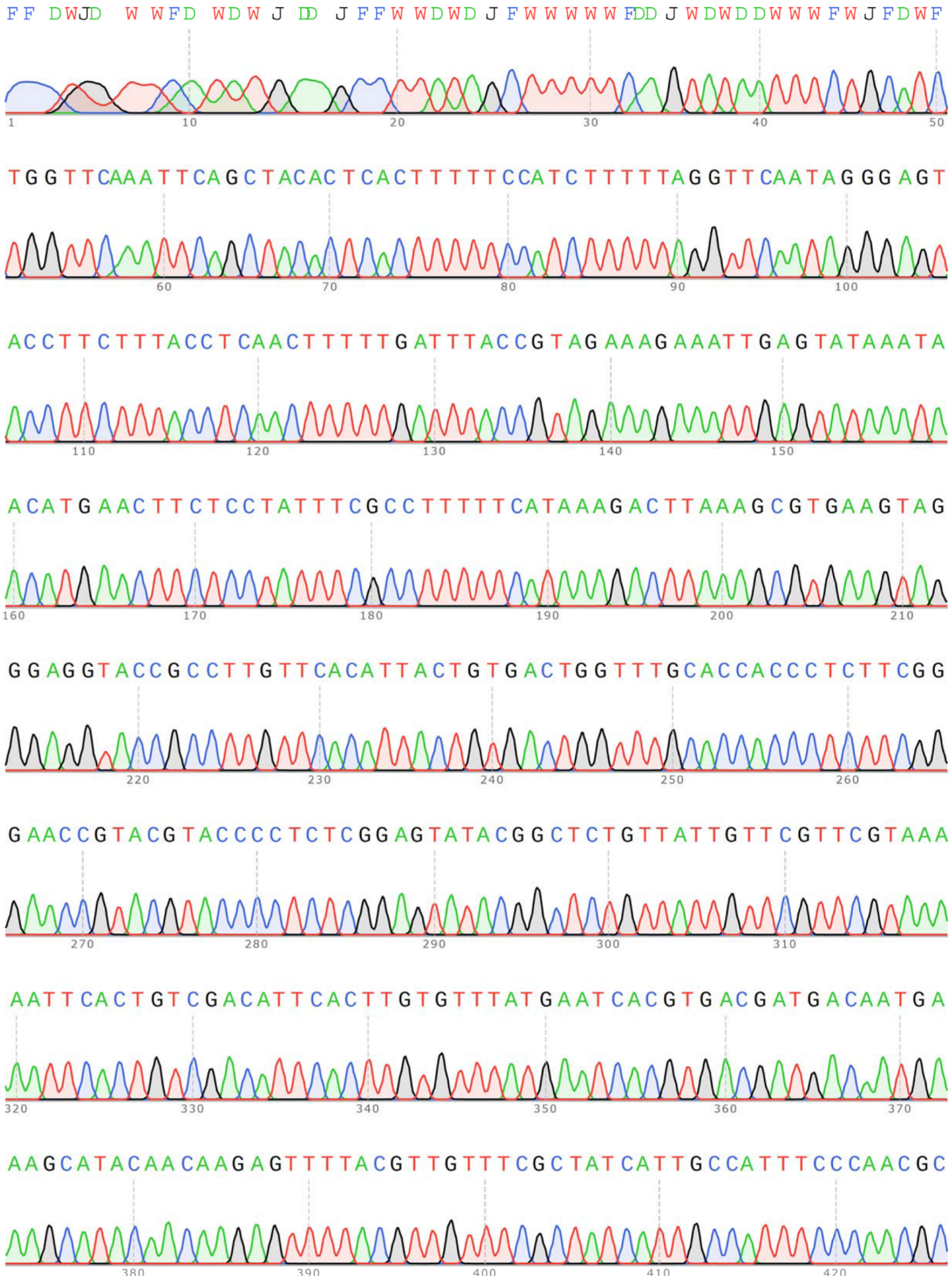

Supplementary Figure S2 DNA sequencing chromatogram for the mutated *atpG* gene in 824-2866KD using atpG-seq-R primer.

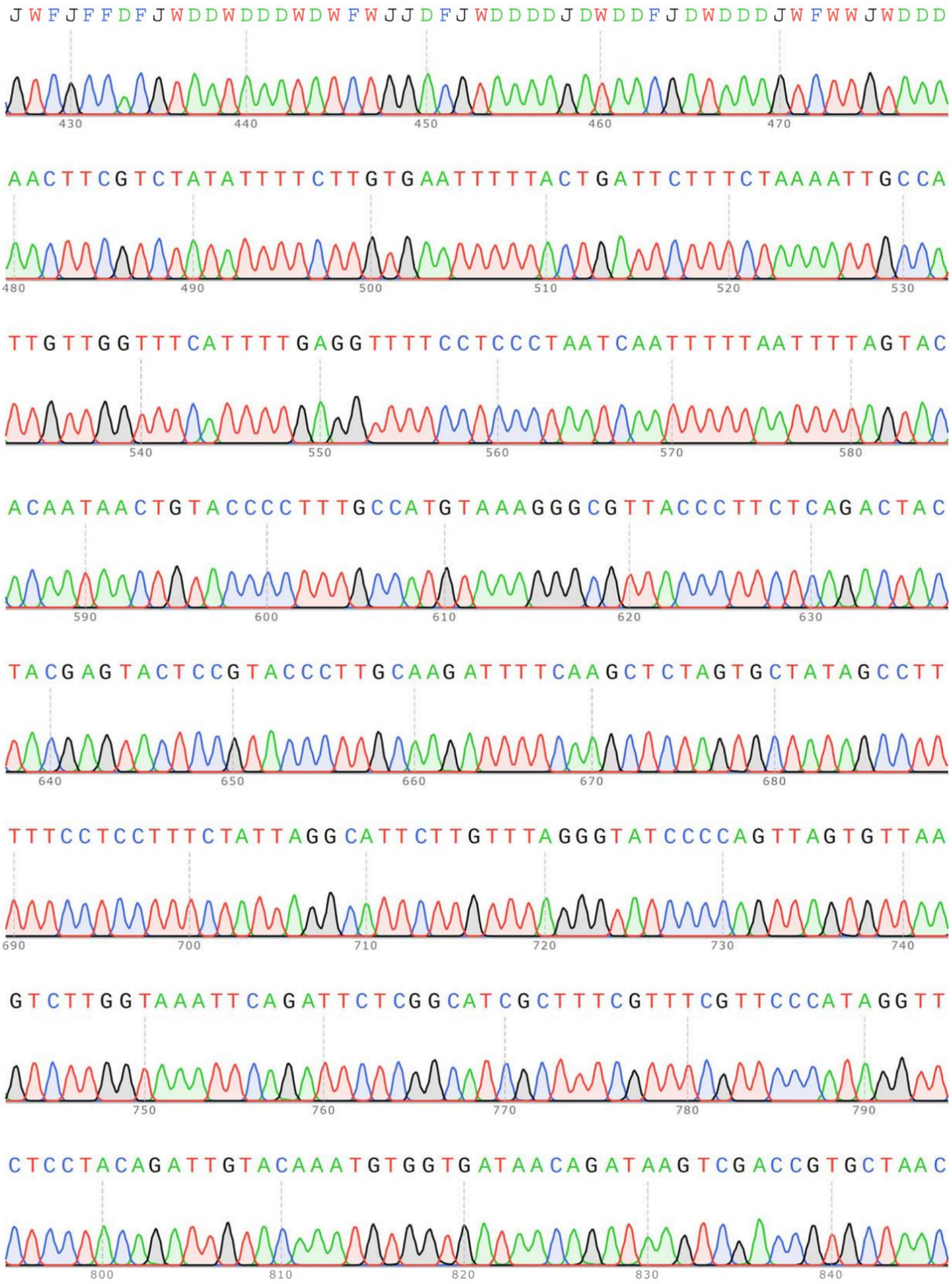

Supplementary Figure S2 (Continued).

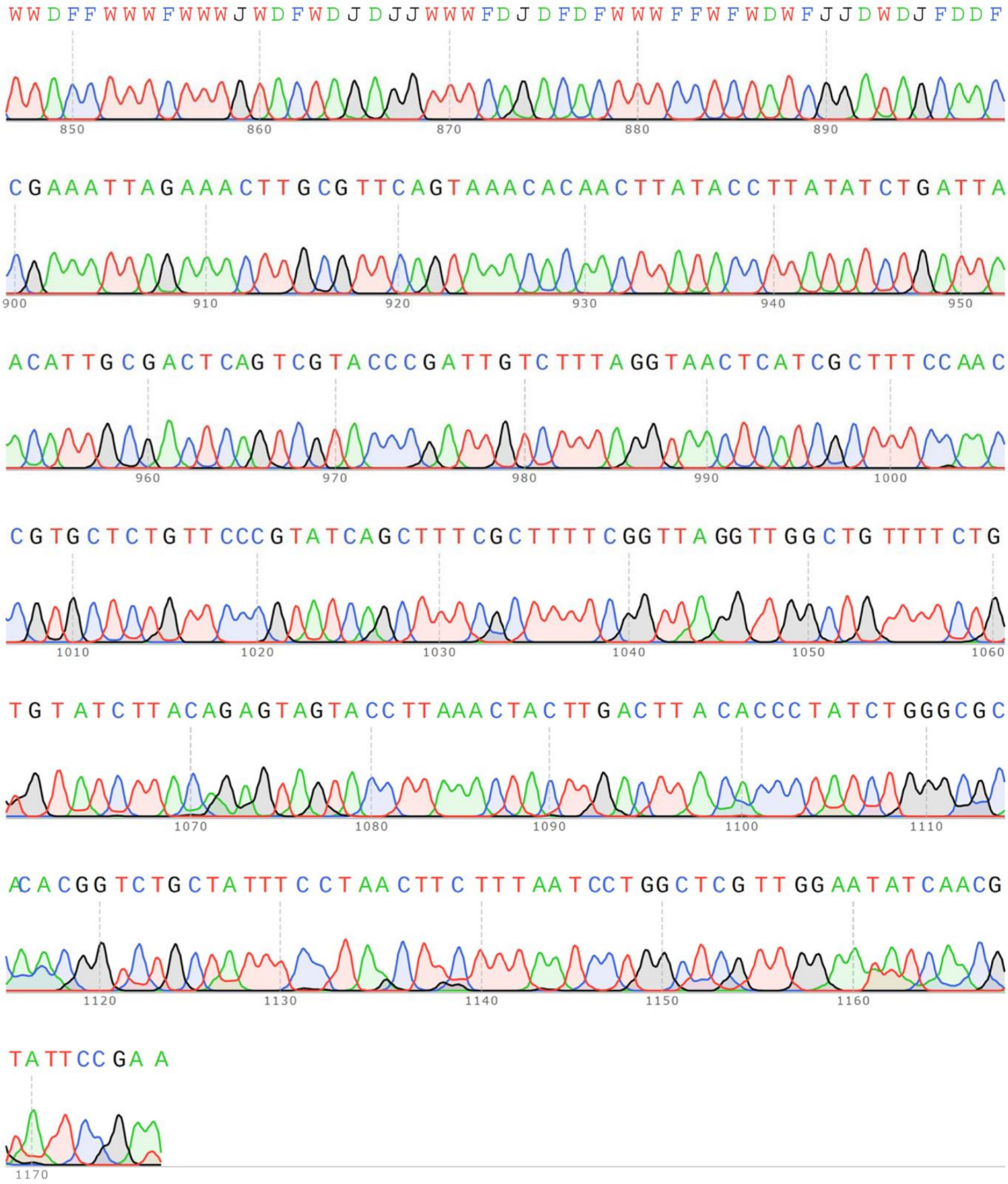

Supplementary Figure S2 (Continued).

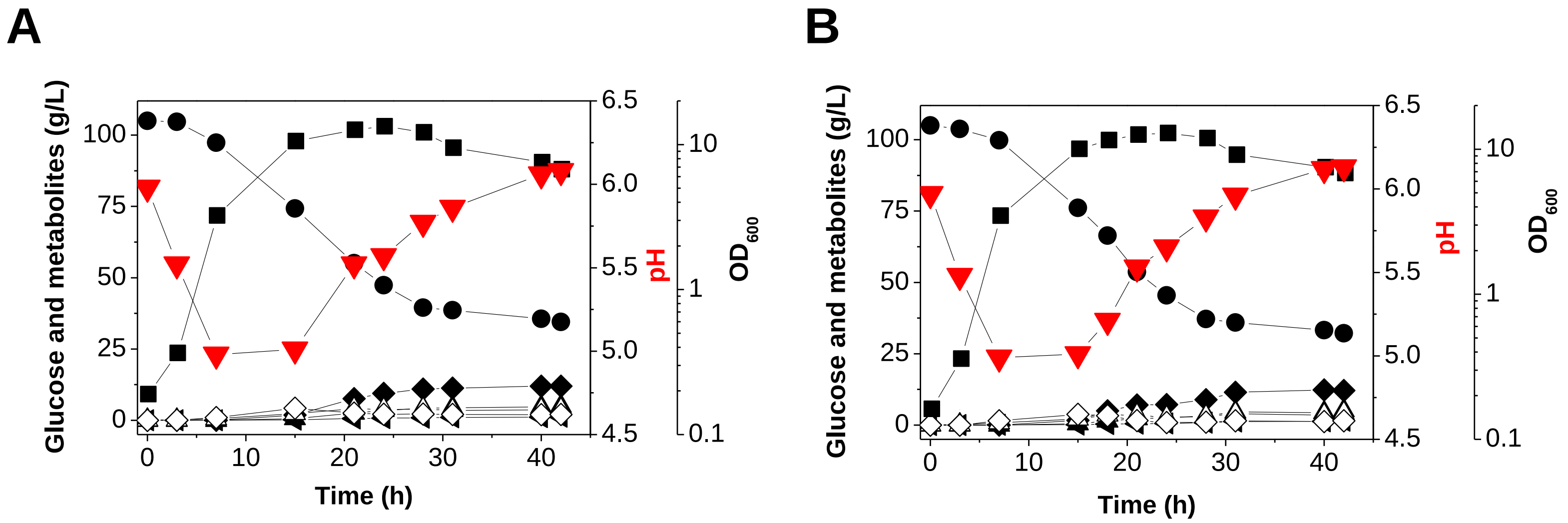

Supplementary Figure S3 (A) Merged version of the batch fermentation profiles of *C. acetobutylicum* 824-2866KD presented in Figure 3. (B) Reproduced batch fermentation profiles of *C. acetobutylicum* 824-2866KD. Symbols: ●, glucose; ■, cell density (OD_{600}); ▼, extracellular pH; △, acetate; ◇, butyrate; ▲, acetone; ◀, ethanol; and ◆, butanol.

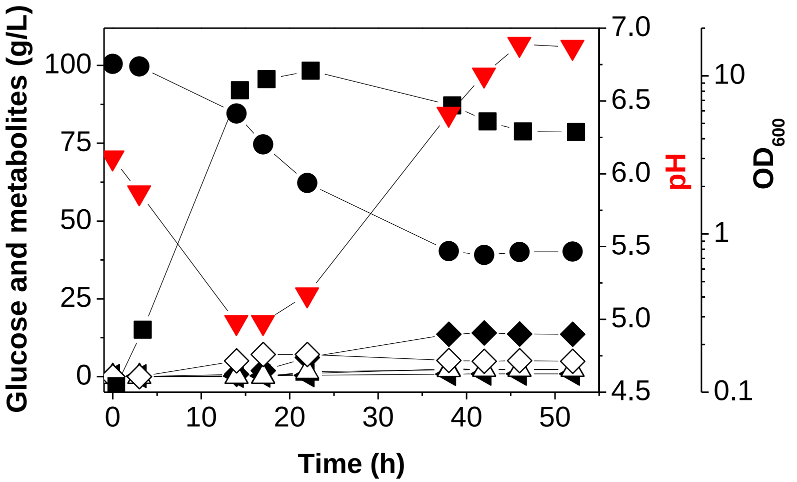

Supplementary Figure S4 Reproduced batch fermentation profiles of *C. acetobutylicum* BEKW-2866KD. Symbols: ●, glucose; ■, cell density (OD_{600}); ▼, extracellular pH; △, acetate; ◇, butyrate; ▲, acetone; ◀, ethanol; and ◆, butanol.

Supplementary Table S1 Oligonucleotides used in this study

Name	Sequences (5’ → 3’)	Reference
**Oligonucleotides**		This study
2866-IBS	AAAAAAGCTTATAATTATCCTTAATAGCCGACCGTGTGCGCCCAGAT AGGGTG	This study
2866-EBS1	CAGATTGTACAAATGTGGTGATAACAGATAAGTCGACCGTGCTAACTTACCTTTCTTTGT	This study
2866-EBS2	TGAACGCAAGTTTCTAATTT CGGTTGCTATCCGATAGAGGAAAGTGTCT	This study
EBS universal	CGAAATTAGAAA CTTGCGTTCA GTAAAC	This study
atpG-F	AGTTCTTCTAGTAAGAGCAACTTATAT	This study
atpG-R	ATATTGAACAGCAGTTATTTAACTTGTTATT	This study
atpG-seq-F	GGTCTTTGTGGAGGTTTTAACG	This study
atpG-seq-R	TTGCCTTAGAATTTAATAACAAGTTAAATAACTG	This study
